# Walking and cycling, as active transportation, and obesity factors in adolescents from eight countries

**DOI:** 10.1186/s12887-022-03577-8

**Published:** 2022-08-30

**Authors:** Gerson Ferrari, Clemens Drenowatz, Irina Kovalskys, Georgina Gómez, Attilio Rigotti, Lilia Yadira Cortés, Martha Yépez García, Rossina G. Pareja, Marianella Herrera-Cuenca, Ana Paula Del’Arco, Miguel Peralta, Adilson Marques, Ana Carolina B. Leme, Kabir P. Sadarangani, Juan Guzmán-Habinger, Javiera Lobos Chaves, Mauro Fisberg

**Affiliations:** 1 Sciences of Physical Activity, Sports and Health School, University of Santiago of Chile (USACH), Santiago, Chile; 2 Faculty of Health Sciences, Universidad Autónoma de Chile, Av. Pedro de Valdivia 425, 7500912 Providencia, Santiago, Chile; 3 Division of Sport, Physical Activity and Health, University of Education Upper Austria, 4020 Linz, Austria; 4 Nutrition Career, Faculty of Medical Sciences, Pontificia Universidad Católica Argentina, AAZ, C1107 Buenos Aires, Argentina; 5 Department of Biochemistry, School of Medicine, Universidad de Costa Rica, San José, 11501-2060 Costa Rica; 6 Department of Nutrition, Diabetes and Metabolism, School of Medicine, Pontificia Universidad Católica, 8330024 Santiago, Chile; 7 Department of Nutrition and Biochemistry, Pontificia Universidad Javeriana, 110231 Bogotá, Colombia; 8 College of Health Sciences, San Francisco de Quito University, Quito, Ecuador; 9 Nutritional Research Institute, La Molina, Lima, Peru; 10 Center for Development Studies, Central University of Venezuela (CENDES/UCV) Bengoa Foundation, Caracas, Venezuela; 11 Pediatric Department, Escola Paulista de Medicina, Federal University of Sao Paulo, Brazil – UNIFESP, São Paulo, Brazil; 12 CIPER, Faculty of Human Kinetics, University of Lisbon, 1499-002 Cruz Quebrada, Lisbon, Portugal; 13 ISAMB, Faculty of Medicine, University of Lisbon, Lisbon, Portugal; 14 Department of Nutrition, School of Public Health, University of São Paulo, São Paulo, Brazil; 15 School of Kinesiology, Faculty of Health and Dentistry, Universidad Diego Portales, 8370057 Santiago, Chile; 16 School of Kinesiology, Faculty of Dentistry and Rehabilitation Sciences, Universidad San Sebastián, Bío Bío, Chile; 17 Sports Medicine and Physical Activity Specialty, Science Faculty, Universidad Mayor, Santiago, Chile; 18 Datrics, Santiago, Chile; 19 Pensi Institute, José Luiz Egydio Setúbal Foundation- Sabará Children’s Hospital, São Paulo, Brazil

**Keywords:** Epidemiology, Active transportation, Physical activity, Neighborhood built environment, Latin America

## Abstract

**Background:**

Evidence has shown that active transportation decreases obesity rates, but considering walking or cycling as separate modes could provide additional information on the health benefits in adolescents. This study aimed to examine the associations between walking and cycling as form active transportation and obesity indicators in Latin American adolescents.

**Methods:**

Population-based study with 671 adolescents (mean age: 15.9 [standard deviation: 0.8] years) from eight countries participating in the Latin American Study of Nutrition and Health/*Estudio Latino Americano Nutrition y Salud* (ELANS). Walking and cycling for active transportation were measured using the International Physical Activity Questionnaire long version. Body mass index, waist circumference, neck circumference, and relative fat mass were used as obesity indicators. Associations were estimated using logistic regression models for the pooled data adjusted for country, sex, age, socio-economic levels, race/ethnicity, leisure-time physical activity and energy intake.

**Results:**

Mean time spent walking and cycling was 22.6 (SD: 33.1) and 5.1 (SD: 24.1) min/day, respectively. The median values were 12.8 (IQR: 4.2; 25.7) and 0 (IQR: 0; 6.2) for walking and cycling. Participants reporting ≥ 10 min/week of walking or cycling for active transportation were 84.2% and 15.5%, respectively. Costa Rica (94.3% and 28.6%) showed the highest prevalence for walking and cycling, respectively, while Venezuela (68.3% and 2.4%) showed the lowest prevalence. There was no significant association between walking for active transportation and any obesity indicator. In the overall sample, cycling for ≥ 10 min/week was significantly associated with a lower likelihood of overweight/obesity based on BMI (OR: 0.86; 95%CI: 0.88; 0.94) and waist circumference (OR: 0.90; 95%CI: 0.83; 0.97) adjusted for country, sex, age, socio-economic level, race/ethnicity, leisure-time physical activity and energy intake compared to cycling for < 10 min/week. There were no significant associations between cycling for active transportation and neck circumference as well as relative fat mass.

**Conclusions:**

Cycling for active transportation was negatively associated with obesity indicators, especially body mass index and waist circumference. Programs for promoting cycling for active transportation could be a feasible strategy to tackle the high obesity rates in adolescents in Latin America.

**Trial registration:**

ClinicalTrials.Gov NCT02226627. Retrospectively registered on August 27, 2014.

## Background

Studies have shown that low levels of physical activity are a risk factor for obesity, and other chronic non-communicable diseases, and increase the likelihood premature death [1]. One important factor that contributes to low physical activity levels is a decrease in the use of active modes of transportation, including walking and cycling [2–4]. This mode of transportation has also been suggested as a feasible approach to increase physical activity levels and reduce the prevalence of child obesity and prevent of chronic disease later in life [5]. Furthermore, cycling for active transportation is associated with a lower risk of cardiovascular disease, cancer, and all cause mortality. Walking for active transportation is associated with a lower risk of cardiovascular disease in adults from Europe [6]. Public health policies and other behavioral-change strategies, therefore, have been encouraging to spend less time on motorized transportation (e.g., cars and other public transportations) [7, 8]. Also, in Latin America, changes in the environmental area and interventions have been adopted favouring time spent walking and cycling [9, 10].

The Latin American region has been showning modifications on population growth, economic development, demographic, and epidemiologic, aspects, resulting in absolut increases in the number of motor vehicles, with marked shifts in travel patterns that are moving away from public transport, walking and cycling, to private motorized vehicles [11, 12]. Rapid, unplanned urban expansion has contributed to environmental and health hazards, including road traffic casualties, air pollution, increased crime and violence and diminished safe spaces for walking and cycling. In the last decade, Latin America has witnessed major urban changes such as an increase in utilities infrastructure and expansion in transport networks, influencing lifestyle changes, obesity and physical activity patterns, that affect active transportation in populations [11, 13]. Most recent data has shown that 19% of Latin American adolescents are considered either overweight or obese, and about 81% do not meet the guidelines for physical activity [13, 14]. For instance, the Active Healthy Kids Global Alliance Physical Activity Report Card for Children and Youth showed that the prevalence of active transportation in Latin America ranges from 20% in Chile to 39% in Colombia and Mexico [15].

Active transportation is a good opportunity to accumulate physical activity and decrease prolonged sitting (i.e. sedentary behaviors) across the lifespan, which potentially influences obesity factors as well as other health outcomes regardless of body fat [16]. Active transportation also reduces the risk for cardiovascular disease, improves the metabolic profile and has many other noted health benefits in youth [17, 18]. However, limited studies have analyzed active transportation in the adolescent population and its positive benefits [17, 19]. Previous studies [18, 19] suggested that active transportation was inversely associated with body mass index (BMI), central obesity, triglyceride levels, blood pressure, and metabolic syndrome. In addition, replacing motorised trips with active transportation also brings numerous co-benefits, such as reduced traffic congestion; improved air quality; and reduced fatalities due to traffic, air pollution, and inactivity [20, 21]. Accordingly walking and cycling for active transportation are particular behaviours of interest for researchers and policy-makers alike.

Epidemiological studies from Latin America have filled numerous gaps showing the association between active transportation and obesity indicators [22–24]. Nevertheless, there is limited evidence on the association between the types of active transportation, i.e., analysed separately, and obesity indicators with a common design across countries using Latin American adolescents. Therefore, there is a need to look for different strategies that can increase walking and cycling and decrease the prevalence of obesity in Latin America. The availability of multi-center cross-sectional survey data from the 2015 “Latin American Study of Nutrition and Health (*Estudio Latinoamericano de Nutrición y Salud*; ELANS)” provides the opportunity to examine how walking and cycling as active transportation are associated with obesity indicators in eight Latin American countries [22, 25]. Moreover, this may provide useful insights for developing public health policies and behavioral-change strategies for the promotion of active transportation. The purpose of the present study was to examine the association between walking and cycling for active transportation and obesity indicators in adolescents from eight Latin American countries.

## Methods

### Study design

We used data from the ELANS [25, 26]. The ELANS is a multi-country cross-sectional survey conducted in eight Latin American countries: Argentina, Brazil, Chile, Colombia, Costa Rica, Ecuador, Peru, and Venezuela. The design was based on a complex and multistage sample, stratified by conglomerates, with all the regions from each country represented, and random selection of the main urban areas within each region according to the proportional probability to size method. Study design, methods, sampling strategy, and exclusion criteria have been published in detail [25, 27] (see https://es.elansstudy.com/).

The ELANS data collection was conducted in the years 2014 to 2015. The ELANS protocol was approved by the Western Institutional Review Board (#20140605) and is registered at ClinicalTrials.gov (#NCT02226627). Each country obtained ethical approval from their home Institutional Review Board. All procedures were performed in accordance with relevant guidelines. Written informed consent obtained from all the ≥ 16 years of age and also from legal guardians/parents of participants < 16 years of age, before commencement of the study.

### Participants

We considered a survey design effect of 1.75 with a limit error of 3.5% and *p* < 0.05, subsequent in a required sample size of 9090 and was expected based on guidance from the National Center for Health Statistics [28]. The participants were selected from Primary Sampling Units areas within each designated city from each country. A number relative to inhabitants weight each of the areas included in the Primary Sampling Units distribution, a representative sample of Secondary Sampling Units was randomly designated using the probability proportional to size method. The sample was stratified by sex, age-range, and socio-economic level. Households within each secondary sampling unit were designated based on a systematic randomization. The total ELANS sample consisted of 9,218 (47.8% men) participants aged 15–65 years, and 671 (58.6% men) adolescents aged 15–17 years with valid data were eligible in the current study. Details about participant sampling and recruitment strategies have been published elsewhere [25–27, 29].

### Active transportation and leisure-time

Participants reported time spent with active transportation and leisure-time physical activity via a 7-day record using the International Physical Activity Questionnaire (IPAQ) long form in Spanish [30]. The IPAQ has been validated through objective methods, i.e., accelerometer (model GT1M) to evaluate total physical activity in adolescents from different countries, with correlations ranging from 0.20 to 0.29 [31].

The IPAQ measures frequency and duration (bouts of ≥ 10 min) of four different domains (i.e., leisure, transport, occupation, household). ELANS study adapted the IPAQ using questions to cover active transportation and leisure-time domains [26, 27], with the following questions:

- For active transportation: (1) *“In the past 7 days, did you walk or use a bicycle for at least 10 consecutive minutes to go from one place to another?”* (With “Yes” and “No” answers); (2) “*In the past 7 days, how many days did you walk or cycle for at least 10 min to go from one place to another?*”; (3) “*How much time did you usually spent in one of these days walking or cycling from one place to another?”*
- For leisure-time: (1) *“In the past 7 days, did you walk or engage in any moderate or vigorous physical activity for at least 10 consecutive minutes?*” (With “Yes” and “No” answers); (2) *“In the past 7 days, how many days did you walk, or engage in moderate or vigorous physical activity for at least 10 min during your leisure-time?*”; (3) “*How much time did you spend walking or in moderate or vigorous physical activity during your leisure time?*”

These questions were asked separately for active transportation and leisure-time. Time (min/week) spent in active transportation and leisure-time was calculated and used for the analysis. Details on the assessment for physical activity using the IPAQ in the ELANS study were detailed in a previous study [25]. Active transportation (walking and cycling) was subsequently dichotomized into < 10 min/week versus ≥ 10 min/week.

### Obesity indicators

Data were collected in each country by trained research assistants during home-visits using standardized procedures [25]. Body weight (kg) was measured with a calibrated electronic scale (Seca®, Hamburg, Germany) with an accuracy of 0.1 kg. Body height (cm) was measured with a portable stadiometer with an accuracy of 0.1 cm. Measurements were taken during inspiration, with the participant’s head positioned in the Frankfort Plane and the base of the stadiometer lightly touching the head [32]. Participants were measured using light clothes with shoes and items removed from the pocket. BMI was calculated and standard deviation (SD) scores were derived using the age- and sex-specific World Health Organization (WHO) growth reference for school-aged adolescents, which were classified into four categories of nutritional / BMI status as follows: underweight (< − 2SD), eutrophic (− 2SD ≥ to ≤ 1SD), overweight (1SD > to ≤ 2SD), and obese (> 2SD) [33].

Waist (WC) and neck circumferences (NC) were measured to the nearest 0.1cm against the skin using an inelastic tape. WC was measured between the lowest rib and the iliac crest after normal expiration [34]. The cut-off points for abdominal obesity was categorized as above or below thresholds (central obesity) based on reference data by sex, age and race/ethnicity for adolescents compiled by Katzmarzyk and colleagues [35]. NC was measured below the larynx, i.e. thyroid cartilage, perpendicular to the long axis of the neck (with the tape in the front of the neck, at the same height as the tape line at the back of the neck) [36]. NC was based on reference data by sex, and age for adolescents [37].

The relative fat mass (RFM) was calculated according to the formula: 64 - (20 × height (m) / WC (m)) + (12 × sex); using sex = 0 for men and 1 for women [38]. The cut-off points defined by Woolcott et al. [39] were used to identify overweight and obesity with RFM ≥25.2 for men and ≥38.7 for women. The RFM is an alternative tool to estimate adolescents total body fat percentage (%). Additionally, evidence suggests that this measurement is more accurate than BMI to provide an estimation of total body fat percentage [38].

### Socio-demographic variables

Age, sex, race/ethinicity, and socio-economic levels (SEL) were collected during home-visits using a standardized questionnaire. Participants were grouped by sex (male and female). SEL was evaluated by questionnaire using a country-dependent format and was based on the legislative requirements or established local standard layouts/criteria. Subsequently, participants were classified as having alow, middle, or high-income on the basis of the low-income measurement, which compares the equivalised per-person income of each country/household with established thresholds for Latin Americans, drawn from national indices used in each country. Race/ethnicity was classified as Caucasian, mixed, black-race (born from parents with different ethnicities), and other (African-American, Asian, Native, and Gypsy). Dietary intake data was obtained from two in-person 24h dietary recall interviews using the automated multiple-pass method [29, 40]. Energy and nutrient content of foods and beverages were determined using the Nutrition Data System for Research Software (NDS-R version 2013) [41]. Energy intake was also used as potential confounder to evaluate the associations between perceived urban environment attributes and obesity indices. Detailed information can be found in a previous publication [26, 42].

### Statistical analysis

Statistical analyses were performed using the software IBM SPSS, v.26. (SPSS Inc., IBM Corp., Armonk, New York,NY, USA). Means (and its SD), median (and its interquartile range: IQR), and percentages were computed, as appropriate, to describe the variables. In this study, we used walking and cycling separately. Walking and cycling for active transportation were stratified into two categories (< 10 min/week, ≥ 10 min/week), indicated as absolute frequencies taking into account the socio-demographic and anthropometric variables and each Latin America country. Chi-square tests (categorical variables) and t test for independent samples (continuous variabels) were carried out to compare the differences between walking and cycling (< 10 min/week [no-active transportation] or ≥ 10 min/week [active transportation]) and the aforementioned variables.

Logistic regression models [odds ratio: OR; 95% confidence interval (95%CI)] were used to verify the association between active transportation (treated as independent variables) and obesity indicators treated as binary dependent variable (0 = underweight/eutrophic and 1 = overweight/obese for BMI and 0 = below threshold and 1 = above threshold for WC, NC and RFM). We performed three logistic regression analyses for each type of active transportation (walking and cycling). The first model was unadjusted. The second model was adjusted for country, sex, age, SEL, and race/ethnicity. The third model was adjusted for leisure-time physical activity and energy intake in addition to the aforementioned variables. Data is presented for the overall ELANS sample. The significance level of 5% (*p* < 0.05) was used and weighting was done according to socio-demographic charateristics and by country [25].

## Results

Overall the response rate for the IPAQ was 99.4%, with a range between 98.5% for Peru to 99.8% for Ecuador. The final sample with complete data was 671 adolescents. Overall, the mean age was 15.9 (SD: 0.8) years. Mean time spent walking and cycling for active transportation were 22.6 (SD: 33.1) and 5.1 (SD: 24.1) min/day. On the other hand, the median values were 12.8 (IQR: 4.2; 25.7) and 0 (IQR: 0; 6.2) for walking and cycling, respectively. Overall, mean and median time spent in leisure-time physical activity, were 44.9 (SD: 49.1) and 25.7 (IQR: 0; 77.1) min/day, respectively. Overall, mean in BMI (kg/m^2^), WC (cm), NC (cm) and RFM were 22.3 (SD: 4.0), 75.3 (SD: 10.8), 33.3 (SD: 3.2), and 24.5 (SD: 9.1). Mean energy intake was 2129.9 (SD: 598.2) kcal/day.

The sample characteristics are presented in Tables 1 and 2. The prevalence of individuals reporting ≥ 10 min/week of walking for active transportation was 84.2%. The prevalence of participants reporting ≥ 10 min/week walking for active transportation ranged from 68.3% (Venezuela) to 94.3% (Costa Rica). There were statistically significant differences (*p* < 0.05) in the proportion of walking (< 10 min/week and ≥ 10 min/week) for active transportation by country, sex, race/ethnicity, BMI, WC, and NC (categorical variables). Alternatively, there was no difference for SEL, and RFM (*p* > 0.05).

**Table 1:** Socio-demographic variables and obesity indicators according to walking for active transportation

Variables	Walking		*p*-value*
	< 10 min/week	≥ 10 min/week	
	N (%)	N (%)	
n (%)	106 (15.8)	565 (84.2)	
**Walking (min/week; mean [SD])**^b^	0.0 (0.0)	188.8 (241.7)	< 0.001
**Country (%)**			< 0.001
Argentina	23 (25.8)	66 (74.2)	
Brazil	22 (17.2)	106 (82.8)	
Chile	8 (11.8)	60 (88.2)	
Colombia	12 (15.8)	64 (84.2)	
Costa Rica	4 (5.7)	66 (94.3)	
Ecuador	4 (6.3)	59 (93.7)	
Peru	7 (7.4)	88 (92.6)	
Venezuela	26 (31.7)	56 (68.3)	
**Sex (%)**			< 0.001
Men	56 (14.2)	337 (85.8)	
Women	50 (18.0)	228 (82.0)	
**Socio-economic level (%)**			0.064
Low	60 (16.9)	294 (83.1)	
Medium	39 (15.5)	212 (84.5)	
High	7 (10.6)	59 (89.7)	
**Race/ethnicity**^a^** (%)**			< 0.001
Caucasian	49 (20.7)	188 (79.3)	
Mixed	43 (13.3)	280 (86.7)	
Black	5 (13.2)	33 (86.8)	
Other	5 (11.6)	38 (88.4)	
**Body mass index (Kg/m**^**2**^**; mean [SD])**^b^	22.3 (4.1)	21.9 (3.6)	0.374
**Categorical (%)**			0.003
Underweight	13 (15.7)	70 (84.3)	
Eutrophic	67 (16.1)	348 (83.9)	
Overweight	20 (16.8)	99 (83.2)	
Obese	6 (11.8)	45 (88.2)	
**Waist circumference (cm; mean [SD])**^b^	75.5 (10.9)	74.4 (10.3)	0.355
**Categorical (%)**			0.002
Below threshold	102 (16.1)	531 (83.9)	
Above threshold	4 (11.4)	31 (88.6)	
**Neck circumference (cm; mean [SD])**^b^	33.4 (3.2)	32.9 (3.2)	0.118
**Categorical (%)**			0.023
Below threshold	55 (17.8)	254 (82.2)	
Above threshold	51 (14.2)	308 (85.8)	
**Relative fat mass (mean [SD])**^b^	24.4 (9.0)	24.7 (9.5)	0.823
**Categorical (%)**			0.178
Below threshold	105 (16.6)	528 (83.4)	
Above threshold	5 (14.4)	30 (85.6)	

Figures as percentages if not stated otherwise;

Chi-square tests (categorical variables) and t test for independent samples (continuous variabels) were applied for comparasion between < 10 min/week and ≥ 10 min/week;

^*^p < 0.05;

^a^*n* = 641;

^b^*n* = 668

**Table 2:** Socio-demographic variables and obesity indicators according to cycling for active transportation

Variables	Cycling		*p*-value*
	< 10 min/week	≥ 10 min/week	
	N (%)	N (%)	
n (%)	567 (84.5)	104 (15.5)	
**Cycling (min/week; mean [SD])**^b^	0.0 (0.0)	236.2 (377.5)	< 0.001
**Country**			< 0.001
Argentina	75 (84.3)	14 (15.7)	
Brazil	101 (78.9)	27 (21.1)	
Chile	59 (86.8)	9 (13.2)	
Colombia	64 (84.2)	12 (15.8)	
Costa Rica	50 (71.4)	20 (28.6)	
Ecuador	52 (82.5)	11 (17.5)	
Peru	86 (90.5)	9 (9.5)	
Venezuela	80 (97.6)	2 (2.4)	
**Sex**			< 0.001
Men	302 (76.8)	91 (23.2)	
Women	265 (95.3)	13 (4.7)	
**Socio-economic level**			0.054
Low	300 (84.7)	54 (15.3)	
Medium	206 (82.1)	45 (17.9)	
High	61 (92.4)	5 (7.6)	
**Race/ethnicity**^a^			< 0.001
Caucasian	202 (85.2)	35 (14.8)	
Mixed	275 (85.1)	48 (14.9)	
Black	36 (94.7)	2 (5.3)	
Other	31 (72.1)	12 (27.9)	
**Body mass index (Kg/m**^**2**^**; mean [SD])**^b^	22.3 (4.0)	21.9 (3.9)	0.464
**Categorical (%)**			< 0.001
Underweight	69 (83.1)	14 (16.9)	
Eutrophic	350 (84.3)	65 (15.7)	
Overweight	102 (85.7)	17 (14.3)	
Obese	44 (86.3)	7 (13.7)	
**Waist circumference (cm; mean [SD])**^b^	75.4 (10.9)	75.2 (10.4)	0.867
**Categorical (%)**			0.004
Below threshold	537 (84.8)	96 (15.2)	
Above threshold	28 (80.0)	7 (20.0)	
**Neck circumference (cm; mean [SD])**^b^	33.2 (3.3)	33.9 (2.9)	0.048
**Categorical (%)**			0.014
Below threshold	255 (82.5)	54 (17.5)	
Above threshold	310 (86.4)	49 (13.6)	
**Relative fat mass (mean [SD])**^b^	25.3 (9.0)	20.1 (8.1)	< 0.001
**Categorical (%)**			0.184
Below threshold	535 (84.5)	98 (15.5)	
Above threshold	25 (71.4)	10 (28.6)	

Figures as percentages if not stated otherwise;

Chi-square tests (categorical variables) and t test for independent samples (continuous variabels) were applied for comparasion between < 10 min/week and ≥ 10 min/week;

^*^p < 0.05;

^a^*n* = 641;

^b^*n* = 668

In regards to cycling for active transportation the overall percentage of participants reporting ≥ 10 min/week was 15.5%: this ranged from 2.4% in Venezuela to 28.6% in Costa Rica. There were statistically significant differences in the proportion of cycling (< 10 min/week and ≥ 10 min/week) for active transportation by country, sex, race/ethnicity, BMI, WC, and NC (categorical variables). There was no difference for SEL, and RFM (Table 2).

Logisitic regression models are presented in Table 3. There were no significant associations between walking for active transportation and obesity indicators (BMI, WC, NC, and RFM) in any of the three models. In all models, ≥ 10 min/week of cycling for active transportation was significantly associated with a lower likelihood for overweight/obesity based on BMI and having a WC above threshold compared to < 10 min/week. For instance, ≥ 10 min/week of cycling was significantly associated with a lower likelihood of overweight/obesity based on BMI (OR: 0.86; 95%CI: 0.88; 0.94) adjusted for country, sex, age, socio-economic level, race/ethnicity, leisure-time physical activity and energy intake versus < 10 min/week. There were no significat associations between cycling and NC and RFM.

**Table 3:** Logistic regression models for the association of walking and cycling for active transportation with obesity indicators

Obesity indicators	Body mass index (0 = underweight/eutrophic, 1 = overweight/obese)		Waist circumference (0 = below threshold, 1 = above threshold)		Neck circumference (0 = below threshold, 1 = above threshold)		Relative fat mass (%) (0 = below threshold, 1 = above threshold)	
	OR (95%CI)	*p*-value	OR (95%CI)	*p*-value	OR (95%CI)	*p*-value	OR (95%CI)	*p*-value
Walking								
< 10 min/week^1^	Ref	0.812	Ref	0.435	Ref	0.206	Ref	0.521
≥ 10 min/week^1^	0.94 (0.58; 1.52)		0.80 (0.44; 1.06)		0.76 (0.50; 1.15)		0.82 (0.30; 1.34)	
< 10 min/week^2^	Ref	0.776	Ref	0.456	Ref	0.205	Ref	0.458
≥ 10 min/week^2^	0.93 (0.57; 1.51)		0.67 (0.23; 1.93)		0.75 (0.50; 1.01)		0.76 (0.38; 1.14)	
< 10 min/week^3^	Ref	0.823	Ref	0.447	Ref		Ref	0.446
≥ 10 min/week^3^	0.94 (0.58; 1.53)		0.66 (0.22; 1.92)		0.21 (0.50; 1.16)	0.215	0.75 (0.45; 1.05)	
Cycling								
< 10 min/week^1^	Ref	< 0.001	Ref	0.002	Ref	0.415	Ref	0.351
≥ 10 min/week^1^	0.76 (0.62; 0.90)		0.65 (0.51; 0.79)		0.70 (0.29; 1.64)		0.89 (0.70; 1.08)	
< 10 min/week^2^	Ref	< 0.001	Ref	0.023	Ref	0.293	Ref	0.286
≥ 10 min/week^2^	0.82 (0.74; 0.90)		0.80 (0.66; 0.94)		0.61 (0.24; 1.52)		0.90 (0.45; 1.35)	
< 10 min/week^3^	Ref	0.003	Ref	0.034	Ref	0.311	Ref	0.323
≥ 10 min/week^3^	0.86 (0.88; 0.94)		0.90 (0.83; 0.97)		0.62 (0.25; 1.54)		0.70 (0.20; 1.20)	

Model 1: unadjusted model;

Model 2: adjusted for country, sex, age, socio-economic level, and race/ethnicity;

Model 3: Model 1 + leisure-time physical activity + energy intake

## Discussion

The present study examined the association between walking and cycling as form of active transportation and obesity indicators in adolescents from eight Latin American countries. The prevalence of individuals reporting ≥ 10 min/week of walking and cycling for active transportation was 84.2% and 15.5%, respectively. Our findings show that cycling ≥ 10 min/week for active transportation significantly reduces the likelihood for overweight/obesity baed on BMI and having a WC above threshold after adjusting for country, sex, age, SEL, race/ethnicity, leisure-time physical activity and energy intake. No significant associations between walking for active transportation and BMI, WC, NC, as well as RFM were found.

Previous studies with small samples usually include cycling and walking as one ‘active’ category. On the other hand, a large nationally representative sample from the United Kingdon [43] showed a significant association between active transport, BMI and %body fat. The ELANS adult study (18-65 years), showed that an increased time spent with active transportation was associated with a lower BMI [22]. Ramírez-Vélez et al. [18] showed that regularly cycling to school was associated with a lower incidence of metabolic syndrome (OR: 0.61; 95%CI: 0.35; 0.99) in Colombian adolescents compared to those using passive transportation. These results corroborates with ours because daily active transportation is an important contributor for total physical activity [18, 44].

The present study suggests that analyzing the two types of active transport together (walking and cycling) may mask greater associations between cycling for active transport and obesity indicators as compared to walking, as cycling requires more energy per minute [45]. Also, in a previous study, cyclists were overall more physically active than pedestrians [6]. Moreover, cycling for active transport was associated with a lower risk of cardiovascular disease, cancer, and all-cause mortality. On the other hand, walking as means of transportation was only associated with a lower risk of adverse cardiovascular disease outcomes [6]. Population health may be improved by policies that increase active transport, predominantly cycling, such as the creation of cycle lanes (also known as ‘ciclovias’), bikesharing schemes, and promoting modal transport.

We found that the prevalence of walking (84.2%) and cycling (15.5%) for active transport was higher compared to United States (10.7% for walking and 1.1% for cycling) [46] and Canada (25% for walking and 6% for cycling) [47]. However, in comparison to European countries like Denmark [48], Finland [49] and Switzerland [50], the prevalence of active commuting in Latin American youth is low: In these countries more than 70% of children and adolescents walk or cycle to school. However, there have been different policies aiming to increase active transport in different Latin American countries [9, 26]. A Brazilian study showed that the existence of ciclovias with <500 yards from home increased the involvement of inhabitants in walking [51]. Studies of such initiatives in Bogotá have shown that users of ciclovias are more likely to comply with physical activity guidelines and have a higher quality of life [52]. Ciclovias are a promising way for increasing physical activity at the inhabitants’ level, and instantaneously addressing other essential urban questions such as equity, quality of life, and healthy physical environments; however safe and wide ciclovias are required through public policies in order to facilitate urban mobility [4].

Based on the lower proportion of adolescents who use walking compared to cycling (84.2% versus 15.5%), our findings suggest that cycling would not be limited as means of active transportation in the Latin America region by vehicle, bike availability and economic development, as there was no clear pattern in the frequency of active transportation. Differences across countries may be attributed to various aspects, including traffic policies (i.e. speed rule), educational equity (i.e. access and facilities in college) and urban planning (i.e. colleges embedded in neighborhood). Several countries in Latin America, e.g., Colombia, have been investing in cycle paths to encourage more cycling as active transportation [9, 52]. Active transportation is an essential compnent not only for increasing physical activity in adolescents, but also for promoting community connectedness and environment safety [53]. In order to encourange active transportation in Latin American countries major coordinative efforts are reqiored, as this behavior is habitually limited by other variables such as perceived traffic, stranger danger or crime safety [26, 54]. Therefore, policy makers, organizations, governments and other key actors should develop multilevel interventions and interdisciplinary strategies for implementing successful programmes, due to the associated and convergent impacts in terms of individual health, traffic, pollution, safety, speed regulations, speed enforcement cameras and opening streets for pedestrians, while highlighting the potential benefits for local the economy and safety for streets users [55, 56].

This study employed a cross-sectional design, precluding inferences about causality. The sample is not nationally representative because only urban areas were analysed. The use of self-report measurements for physical activity should be considered for the interpretation of these results. Caution with the interpretation of the results using the IPAQ is also warranted due tocultural differences. This could reduce the probability of significant associations with anthropometric measurements. The temporality of when data were collected (i.e., 2014–2015) relative to the release of the active transportation and obesity rates are a limitation. It is possible that the movement behaviors of adolescents across Latin America have changed over this time period. The race/ethnicity and obesity indicators had substantial missing data. However, missing data were not related to active transportation. Alternatively, strengths of this study includes: (i) examination of walking and cycling as active transportation in eight Latin American countries, (ii) a large sample size using a common design and comparable methods, which are scarce among Latin American epidemiology studies. Due to the fact that this study provides a unique Latin American dataset, cross-country comparisons, however, are not possible.

## Conclusion

The study showed that cycling for active transportation was associated with lower BMI and WC in the ELANS sample, independent of country, sex, age, SEL, race/ethnicity, leisure-time physical activity and energy intake. There was no significant association between walking for active transportation and obesity indicators.

Cycling as active transportation, therefore, should be considered as public health policy and integrated in behavioral-change strategies to encourage people to incorporate this into their routines, which in turn, might help with increasing levels of physical activity and decreasing obesity levels in the Latin American population.

## Data Availability

The datasets generated and/or analyzed during the current study are not publicly available due the terms of consent/assent to which the participants agreed but are available from the corresponding author on reasonable request. Please contact the corresponding author to discuss availability of data and materials.
